# Genome-Wide Analysis of Soybean JmjC Domain-Containing Proteins Suggests Evolutionary Conservation Following Whole-Genome Duplication

**DOI:** 10.3389/fpls.2016.01800

**Published:** 2016-12-05

**Authors:** Yapeng Han, Xiangyong Li, Lin Cheng, Yanchun Liu, Hui Wang, Danxia Ke, Hongyu Yuan, Liangsheng Zhang, Lei Wang

**Affiliations:** ^1^College of Life Sciences, Xinyang Normal UniversityXinyang, China; ^2^Institute for Conservation and Utilization of Agro-bioresources in Dabie Mountains, Xinyang Normal UniversityXinyang, China; ^3^Center for Genomics and Biotechnology, Haixia Institute of Science and Technology, Fujian Agriculture and Forestry UniversityFuzhou, China; ^4^Key Laboratory of Genetics, Breeding and Multiple Utilization of Corps (Fujian Agriculture and Forestry University), Ministry of Education, Fujian Provincial Key Laboratory of Haixia Applied Plant Systems Biology, Fujian Agriculture and Forestry UniversityFuzhou, China

**Keywords:** soybean (*Glycine max* L.), *JmjC* gene family, genome-wide analysis, phylogeny, gene structure, expression pattern

## Abstract

Histone modifications, such as methylation and demethylation, play an important role in regulating chromatin structure and gene expression. The JmjC domain-containing proteins, an important family of histone lysine demethylases (KDMs), play a key role in maintaining homeostasis of histone methylation *in vivo*. In this study, we performed a comprehensive analysis of the *jumonji C* (*JmjC*) gene family in the soybean genome and identified 48 *JmjC* genes (*GmJMJs*) distributed unevenly across 18 chromosomes. Phylogenetic analysis showed that these JmjC domain-containing genes can be divided into eight groups. *GmJMJs* within the same phylogenetic group share similar exon/intron organization and domain composition. In addition, 16 duplicated gene pairs were formed by a *Glycine*-specific whole-genome duplication (WGD) event approximately 13 million years ago (Mya). By investigating the expression profiles of these gene pairs in various tissues, we showed that the expression pattern is conserved in the polyploidy-derived JmjC duplicates, demonstrating that the majority of *GmJMJs* were preferentially retained after the most recent WGD event and suggesting important roles for demethylase duplications in soybean evolution. These results shed light on the evolutionary history of this family in soybean and provide insights into the *JmjCs* which will be helpful to reveal their functions in controlling soybean development.

## Introduction

Histone methylation and demethylation have important roles in regulating transcription, genome integrity, and epigenetic inheritance (Klose et al., 2006; Klose and Zhang, 2007; Liu et al., 2010). Histone methylation can occur at various lysine and arginine residues, including K4, K9, K27, K36, and K79 in histone H3 and K20 in histone H4 (Allis et al., 2007). Histone methylation, which is mainly catalyzed by protein families that contain PRMT and SET domains, can have both activating and repressive effects on chromatin function (Ahmad and Cao, 2012; Zhang and Ma, 2012). Two kinds of demethylase are involved in the homeostasis of methylation in organisms. Lysine Specific Demethylase 1 (LSD1) was the first histone demethylase identified and is a member of the flavin-dependent amine oxidase family (Lee et al., 2005; Metzger et al., 2005; Chen et al., 2011). Genes in the second class of histone demethylases have a JmjC domain with which they catalyze histone lysine demethylation through oxidative reactions dependent on ferrous ion (Fe(II)) and α-ketoglutarate (α-KG) (Elkins et al., 2003; Trewick et al., 2005).

Plant JmjC proteins are known to play important roles in regulating epigenetic processes and in growth and development (Klose et al., 2006; Kouzarides, 2007). Many members of the *JmjC* gene family from different plant species have been characterized. In *Arabidopsis*, AtJMJ11/ELF6 (EARLY FLOWERING 6) is a repressor in the photoperiodic flowering pathway, and its loss-of-function mutation causes early flowering (Noh et al., 2004; Yu et al., 2008). Its relative, AtJMJ12/REF6 (RELATIVE OF EARLY FLOWERING 6) has an opposite effect in the regulation of flowering time (Noh et al., 2004; Yu et al., 2008; Lu et al., 2011a). Loss-of-function mutation of REF6 leads to increased expression of the flowering repressor FLC (FLOWERING LOCUS C) and hence late flowering (Lu et al., 2011a). In addition, AtJMJ14, an active histone H3K4 demethylase (Lu et al., 2010; Yang et al., 2010), was also implicated in preventing early flowering by repressing the expression of *FLOWERING LOCUS T* (*FT*) and its homologs. Recently, Ning reported that AtJMJ14 associates with two NAC transcription factors, NAC050 and NAC052, and co-occupies hundreds of common target genes, resulting in H3K4 demethylation and transcriptional repression (Ning et al., 2015). Apart from controlling flowering time, there is also evidence that AtJMJ14 functions in RNA silencing and cell-to-cell movement of an RNA silencing signal (Lu et al., 2010). The histone H3K9 demethylase AtJMJ25/IBM1 (INCREASE IN BONSAI METHYLATION 1) (Wang et al., 2013; Shen et al., 2014a) protects genes from CHG (H represents A, T, or G) hypermethylation by CMT3 (CHROMOMETHYLASE 3). Gain-of-function mutants of *AtJMJ15* showed enhanced salt tolerance, in contrast with increased salt sensitivity in the loss-of-function mutant (Shen et al., 2014a). In addition, *AtJMJ14* and *AtJMJ15* have also been shown to be involved in the control of flowering time (Yang et al., 2012). AtJMJ30/JMJD5, an evening-expressed gene, is the sole AtJMJ protein to show a robust circadian rhythm of expression (Mockler et al., 2007; Michael et al., 2008; Jones and Harmer, 2011; Lu et al., 2011b). The role of *AtJMJ30* as a genetic regulator of period length in the *Arabidopsis* circadian clock was confirmed by analysis of loss- and gain-of-function mutants (Lu et al., 2011b). In tomato, a similar role is played by *JMJ524*, which standardizes the circadian clock and also alters GA response to regulate stem elongation (Li et al., 2015). In *Medicago truncatula, MtJMJC5* (Medtr4g066020), an ortholog of *AtJMJ30/JMJD5*, may play a role in epigenetic regulation of the link between the circadian clock and cold signaling (Shen et al., 2016). In rice, OsJMJ705 is a biotic stress-responsive H3K27me2/3 demethylase that may remove H3K27me3 from marked defense-related genes and increase their basal and induced expression during pathogen infection (Li et al., 2013). *OsJMJ706*, encodes a heterochromatin-associated H3K9 demethylase, is reported to involve in the regulation of flower development in rice (Sun and Zhou, 2008).

Soybean [*Glycine max* (L.) Merr.] is one of the most economically important crop species in the world. Its genome has undergone two rounds of whole-genome duplication (WGD; Schlueter et al., 2004; Schmutz et al., 2010; Vanneste et al., 2014; Liu et al., 2015); one occurred approximately 59 Mya and was shared by other legumes such as *Medicago* and *Lotus*, while the other was specific to *Glycine* and occurred around 13 Mya. Thus, about 75% of the genes in the soybean genome have multiple paralogs (Schmutz et al., 2010; Severin et al., 2011; Singh and Jain, 2015), making it an excellent model for studying the evolution of duplicate genes following polyploidy. Here, we systemically identify the *JmjC* gene family members in soybean (*G. max*), *Medicago* (*M. truncatula*), and *Lotus* (*Lotus japonicus*) and subsequently analyze the evolutionary relationships between these genes among the three legumes, *Oryza sativa*, and *Arabidopsis*. In addition, we study the *GmJMJs* in further detail, including subfamily classification, gene structures, chromosomal distribution, duplication patterns, conserved residues, and expression profiling. We propose that demethylases exhibit conservative functions through duplication events. Our data will facilitate future studies to elucidate the exact biological functions of the *GmJMJs*.

## Materials and methods

### Identification of JmjC domain-containing proteins in soybean and other legumes

The *G. max* 2.0 genome database (https://phytozome.jgi.doe.gov/pz/#) was searched to identify JmjC domain-containing proteins using Basic Local Alignment Search Tool algorithms (BLASTP) with a threshold of *e*-value < 1e-10, using the published *Arabidopsis* (21) and *O. sativa* (20) JmjC domain-containing protein sequences (Table S1) as queries (Huang et al., 2016). All obtained protein sequences were examined for the presence of the JmjC (PF02373, SM00558) domain using the Hidden Markov Model (HMM) of Pfam (Finn et al., 2016) (http://pfam.sanger.ac.uk/search), and SMART (Letunic et al., 2015) (http://smart.embl-heidelberg.de/). Sequences with obvious errors and/or JmjC domain length of <90 amino acids were removed manually. Following the same approach, putative *M. truncatula* and *L. japonicus* JmjC domain-containing proteins were identified from Phytozome v10 (https://phytozome.jgi.doe.gov/pz/portal.html) and the *L. japonicus* genome assembly build 3.0 (http://www.kazusa.or.jp/lotus/), respectively.

### Phylogenetic analysis

**Figure 1:**
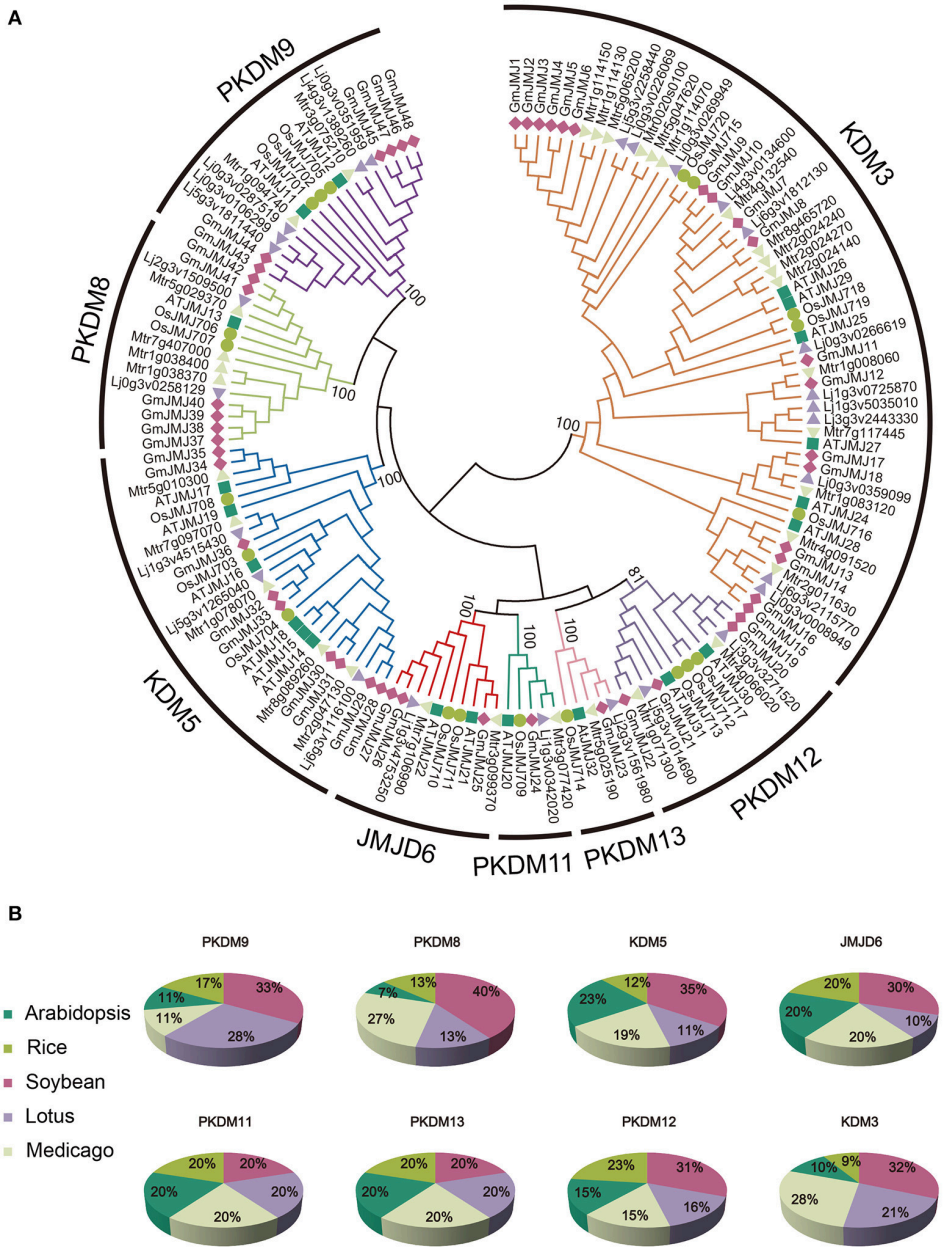
**Phylogenetic relationship and distribution of JmjC-domain containing proteins from five plant species. (A)** Phylogenetic relationship of JmjC-domain containing proteins among soybean, *Medicago, Lotus*, rice, and *Arabidopsis*. Both multiple sequences alignment and phylogenetic tree were performed by MEGA6.0. The value at the nodes represents bootstrap values from 1000 replicates. Different groups were shown by different color. **(B)** Percentage representation of JmjC-domain containing proteins across the five plant species within each group. Colors correspond to the plant taxa as listed in the left.

Multi-species phylogenetic tree was constructed using MEGA 6.0 (Tamura et al., 2013) with the Neighbor-Joining (NJ) method, and bootstrap analysis was conducted using 1000 replicates with the p-distance model. The JmjC domain alone was used to set up the phylogenetic tree and define the groups (Figure S1). And then with the aim to obtain a better phylogeny within each group, we added additional conserved domains to JmjC domain (Table S2) to construct the phylogenetic tree (Figures 1A, **4A**). Multiple sequences alignments were performed using ClustalW with default parameters in MEGA 6.0.

### Chromosomal locations and gene structure of *JmjC* genes

The locations of the JmjC domain-containing genes on the soybean chromosomes were plotted using the MapChart software. The location information of each JmjC domain-containing gene on each chromosome was determined from the soybean genome annotation file (Gmax_275_Wm82.a2.v1.gene.gff3). The blocks regarded as recent duplications were obtained from SoyBase (Grant et al., 2010) (http://www.soybase.org/). The presence of introns and exons was also annotated according to the soybean genome annotation file. Schematic diagrams were pictured by using GSDS2.0 (Gene Structure Display Server http://gsds.cbi.pku.edu.cn/).

### Conserved domains and conserved residues in the JmjC domain-containing proteins

To explore the full-length sequences of JmjC domain-containing proteins, NCBI CDD (Marchler-Bauer et al., 2015) (http://www.ncbi.nlm.nih.gov/cdd/), SMART (http://smart.embl-heidelberg.de/), and Pfam (http://pfam.xfam.org/) were performed with default parameters to search for conserved domains. To identify conserved amino acid residues for interaction with co-factors, the sequences of JmjC domain were aligned using the DNAMAN software.

### Expression analysis of soybean *JmjC* genes

To determine the expression patterns of the *JmjC* genes in soybean tissues, transcriptome data was downloaded from the NCBI Short Read Archive database under the following accession numbers: SRX474427, SRX474441, SRX474445, SRX474430, SRX474431, SRX474433, SRX474432, SRX474439, SRX474442, SRX474419, SRX474428, SRX474440, SRX474443, SRX474424, SRX474423, SRX474422, SRX474434, SRX474436, SRX474437, SRX474416, SRX474435, SRX474438, SRX474421, SRX474420, SRX474446, SRX474444, SRX474426, and SRX474429 (Shen et al., 2014b). Transcriptome analysis was performed to identify expression patterns in representative tissues, including roots, cotyledons, stems, shoot meristems, leaf buds, leaves, flowers, pods, pod and seeds, and seeds (Table S3). Finally, heatmaps of *GmJMJ* expression were produced using the pheatmap packages in R.

### Calculation of Ka/Ks-values and evaluation divergence time

To investigate whether positive Darwinian selection was involved in *GmJMJ* divergence following duplication and to estimate the date of the duplication pairs, the non-synonymous (Ka) and synonymous substitution (Ks) rate ratios of the paralog pairs were calculated using the YN00 method of the PAML program (Yang, 2007). Based on a rate of 6.1 × 10^−9^ substitutions per site per year, we calculated the divergence time (*T*) as *T* = Ks/(2 × 6.1 × 10^−9^) × 10^−6^ Mya (Lynch and Conery, 2000).

## Results

### Identification of *JmjC* gene family in soybean

Using the combined methods, we identified a total of 48 *GmJMJs*, which is more than twice the number found in *Arabidopsis* (21) or rice (20) (Lu et al., 2008). To better understand the expansion and evolutionary history of *GmJMJs*, the same methods were used to search for *JmjC* genes in two other legumes, *Medicago* and *Lotus*. We identified 33 *Medicago* and 27 *Lotus JmjC* genes, which is still less than the number found in soybean.

A variety of information about *GmJMJs*, such as different version of gene codes, gene length, isoelectric point (pI), and molecular weight (Mw) and so on, were listed in Table S4. For example, the identified *GmJMJs* encode proteins ranging from 284 (GmJMJ2) to 1831 (GmJMJ35) amino acids, with the isoelectric point (pI) varying from 4.91 (GmJMJ23) to 9.25 (GmJMJ37) and the molecular weight (Mw) varying from 32.2 kD (GmJMJ2) to 209.2 kD (GmJMJ35). *GmJMJ1* was excluded from further analyses, as there is no annotation data available for it.

### Phylogenetic analysis of *JmjC* genes in soybean

According to the phylogenetic analysis (Figure 1A), the *JmjC* genes can be divided into eight groups: PKDM9, PKDM8, KDM5, JMJD6, PKDM11, PKDM13, PKDM12, and KDM3. KDM3 has the most members, with 57 homologous *JmjC* genes, and KDM5 is the second largest group, containing 26 *JmjC* genes. The smallest clades are PKDM11 and PKDM13, which both consist of only five *JmjC* genes, one from each species. In general, most of the clades include genes from all five species, although the clades are also enriched in particular species. For example, doubled *GmJMJ* pairs are sister genes to *AtJMJ28* in a clade which also includes doubled genes from the other legumes, forming a cluster of several legume *JMJ* genes with a single *AtJMJ* gene. Likewise, there is a larger of percentage of soybean (40%) than *Arabidopsis* (7%) genes in PKDM8 (Figure 1B). These findings indicate that different levels of gene duplication or lose may have been occurred among the five species after the divergence of eudicot and monocot.

The phylogenetic relationships of *AtJMJ30, OsJMJ717*, and *MtJMJC5* (Mtr4g066020) in PKDM12 are consistent with a recent report (Shen et al., 2016). Shen et al. showed that *MtJMJC5* is involved in regulating circadian rhythm (Shen et al., 2016). And recently, Li et al also reported that JMJ524, consistent with its counterparts AtJMJ30, is also involved in a circadian clock response in tomato (Li et al., 2015). Based on the phylogenetic analysis we hypothesis that soybean orthologs of *MtJMJC5* and *JMJ524 (GmJMJ19* and *GmJMJ20)* may also play similar roles in rhythm regulation.

### Chromosome location and duplication of *GmJmJ* genes

**Figure 2:**
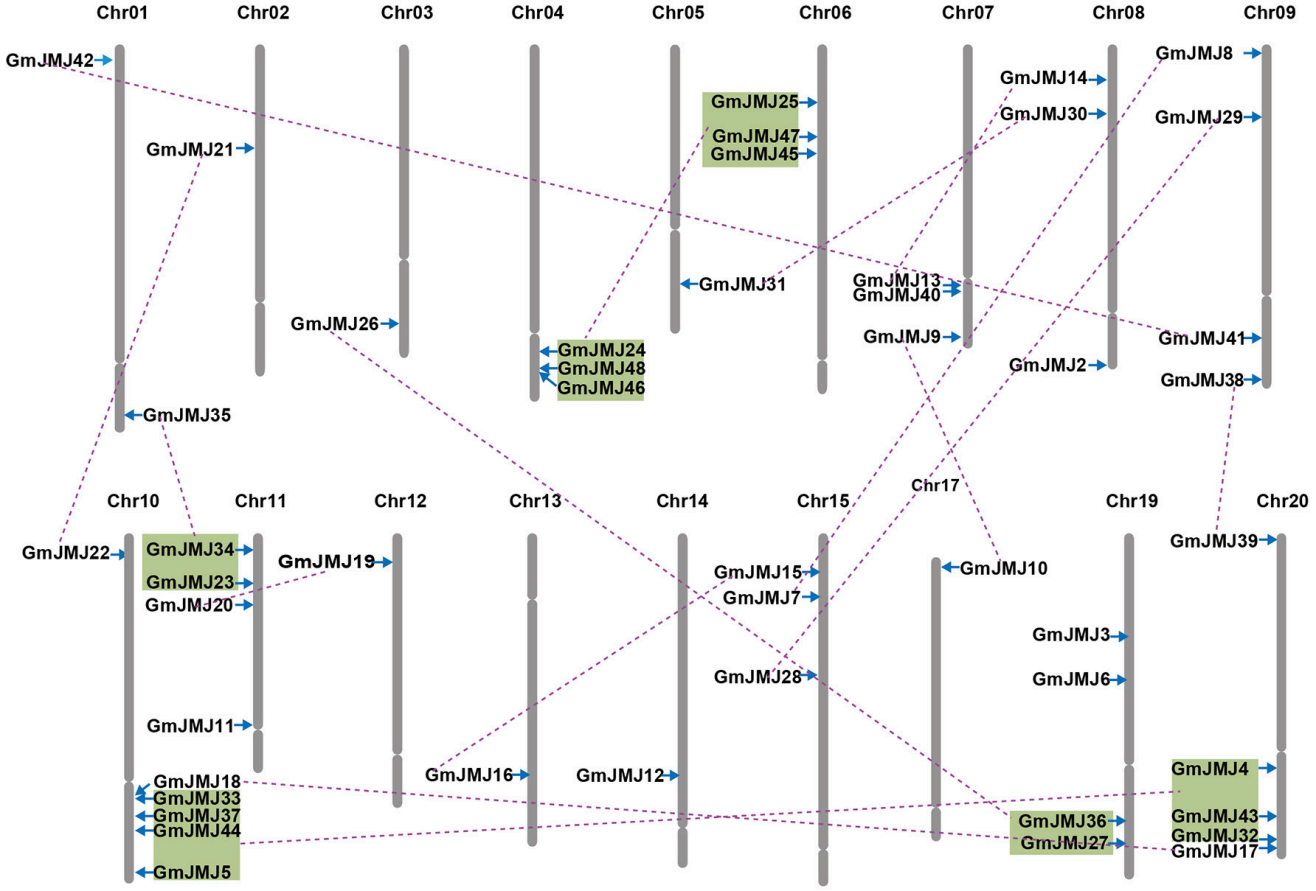
**Chromosomal locations, region duplication, and predicted cluster for soybean JmjC genes**. The chromosome number is indicated above each chromosome. Throat on the chromosomes indicate the position of centromere. Only those chromosomes bearing JmjC genes (18) were represented. Segmental duplicated genes are indicated by purple lines. Colored boxes indicate the gene blocks generated by the most recently WGD event. The figure was produced and modified using the MapChart and Adobe Illustrator, respectively.

**Figure 3:**
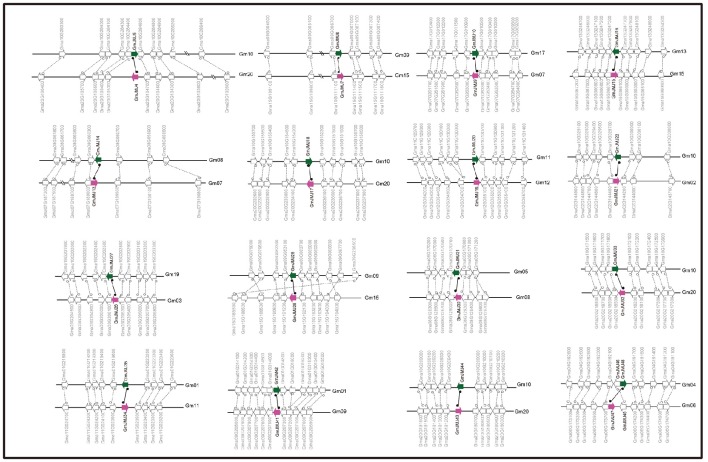
**Collinearity analysis of duplicated gene pairs formed by Glycine-specific WGD event**. Green and purple arrows represented duplicated JmjC gene pairs. The direction of the arrow indicates the position of the gene in the positive (to left) and negative (to right) chain of DNA.

Compared to other species, soybean has an extensively expanded *GmJMJ* family with more than twice as many *JmjCs* as rice and *Arabidopsis* (Table 1). We carried out a comprehensive analysis of the *GmJMJs* with the aim of understanding their duplication status and identifying duplicated gene pairs. First, *GmJMJ* pairs located in a pair of paralogous blocks formed by *Glycine*-specific WGD were considered as candidate duplicate gene pairs. As shown in Figure 2, all 47 *GmJMJs* (except *GmJMJ1*) were randomly located on 18 of the 20 soybean chromosomes. For example, chromosome 10 possesses six *GmJMJs*, chromosomes 4, 6, 7, 8, and 15 each contain three *GmJMJs*, and chromosome 2, 3, 5, 12, 13, 14, and 17 each have only one *GmJMJ*. In total, we found that a large proportion (41 of 47) of the *GmJMJs* (linked by purple lines in Figure 2) was distributed preferentially in duplicated blocks. These 41 genes be considered as candidates of the most recent *Glycine*-specific WGD and used in the next analysis. Second, close phylogenetic relationships have been shown among all candidate *GmJMJs* (Figure S2). Third, duplication types of all *GmJMJs* have been obtained by MCscanX programs (Wang et al., 2012) (Table S5). Fourth, collinearity analysis (Figure 3) was carried out among each candidate duplicated gene pairs. The candidate pairs were considered as created by the recent Glycine-specific WGD duplications with at least three paralogous gene pairs along the flanking regions. In conclusion, we identified that 16 *GmJMJ* pairs were formed by the most recent *Glycine*-specific WGD (Table 2).

**Table 1:** **JmjC gene distribution among species that were used in this study**.

**Species name**	**Abbr**.	**PKDM9**	**PKDM8**	**KDM5**	**JMJD6**	**PKDM11**	**PKDM13**	**PKDM12**	**KDM3**	**All**	**Genome size (mb)**	**Chromosomes**
*Glycine max*	Gma	6	6	9	3	1	1	4	18	48	1100	40
*Lotus japonicus*	Lj	5	2	3	1	1	1	2	12	27	472	12
*Medicago truncatula*	Mtr	2	4	5	2	1	1	2	16	33	500	16
*Arabidopsis thaliana*	At	2	1	6	2	1	1	2	6	21	125	10
*Oryza sativa*	Os	3	2	3	2	1	1	3	5	20	466	24

**Table 2:** **Divergence between JmjC gene pairs in soybean**.

**Gene1**	**Gene2**	**Ka**	**Ks**	**Ka/Ks**	**Duplication date (Mya)**	**Duplication type**
GmJMJ4	GmJMJ5	0.101	0.186	0.541	15.3	WGD or segmental
GmJMJ7	GmJMJ8	0.072	0.189	0.381	15.5	WGD or segmental
GmJMJ9	GmJMJ10	0.046	0.076	0.069	6.2	WGD or segmental
GmJMJ13	GmJMJ14	0.034	0.090	0.380	7.4	WGD or segmental
GmJMJ15	GmJMJ16	0.026	0.068	0.381	5.6	WGD or segmental
GmJMJ17	GmJMJ18	0.018	0.169	0.104	13.8	WGD or segmental
GmJMJ19	GmJMJ20	0.028	0.147	0.191	12.1	WGD or segmental
GmJMJ21	GmJMJ22	0.072	0.126	0.574	10.3	WGD or segmental
GmJMJ26	GmJMJ27	0.043	0.171	0.250	14.0	WGD or segmental
GmJMJ28	GmJMJ29	0.036	0.103	0.346	8.4	WGD or segmental
GmJMJ30	GmJMJ31	0.035	0.077	0.458	6.3	WGD or segmental
GmJMJ32	GmJMJ33	0.026	0.074	0.359	6.0	WGD or segmental
GmJMJ34	GmJMJ35	0.024	0.101	0.241	8.3	WGD or segmental
GmJMJ41	GmJMJ42	0.026	0.104	0.250	8.5	WGD or segmental
GmJMJ43	GmJMJ44	0.030	0.092	0.333	7.5	WGD or segmental
GmJMJ47	GmJMJ48	0.045	0.130	0.349	10.7	WGD or segmental

Based on the divergence rate of 6.1 × 10^−9^ synonymous mutations per synonymous site per year which has been proposed for soybean (Lynch and Conery, 2000), among the 48 *JmjCs* in soybean, 73% (35 of 48) represented WGD/segmental duplication genes. Ks-value was calculated for estimating the separation time of each paralogous gene pair. All Ks-values ranged from 0.068 to 0.18, which was consisted with whole genome duplication events at round 13 Mya. In addition, our divergence time analyses showed that duplications among 16 paralogous pairs occurred between 5.6 and 15.5 Mya, with an average of 9.7 Mya (Table 2).

The history of selection acting on coding sequences can also be measured based on the ratio of non-synonymous to synonymous substitutions (Ka/Ks) (Li et al., 1981). Ka and Ks can be estimated using a number of substitution models and methods, and the estimates are sensitive to these choices and other complications such as the GC content of the sequences and their genomic context (Bustamante et al., 2002). Ka is usually much smaller than Ks, so a pair of sequences will have Ka/Ks << 1 if both sequences have been under purifying selection, Ka/Ks < 1 if one sequence has been under purifying selection but the other drifting naturally, and in rare, Ka/Ks > 1 when both sites are under positive seletion (Juretic et al., 2005). As shown in Table 2, the average Ka/Ks-values of the *GmJMJ* gene pairs were 0.36. Five paralog pairs have small Ka/Ks ratios (< 0.3), most Ka/Ks ratios in the range from 0.3 to 0.7, and none of them > 1.

### Exon–intron structure and domain architecture of *GmJmJ* genes

**Figure 4:**
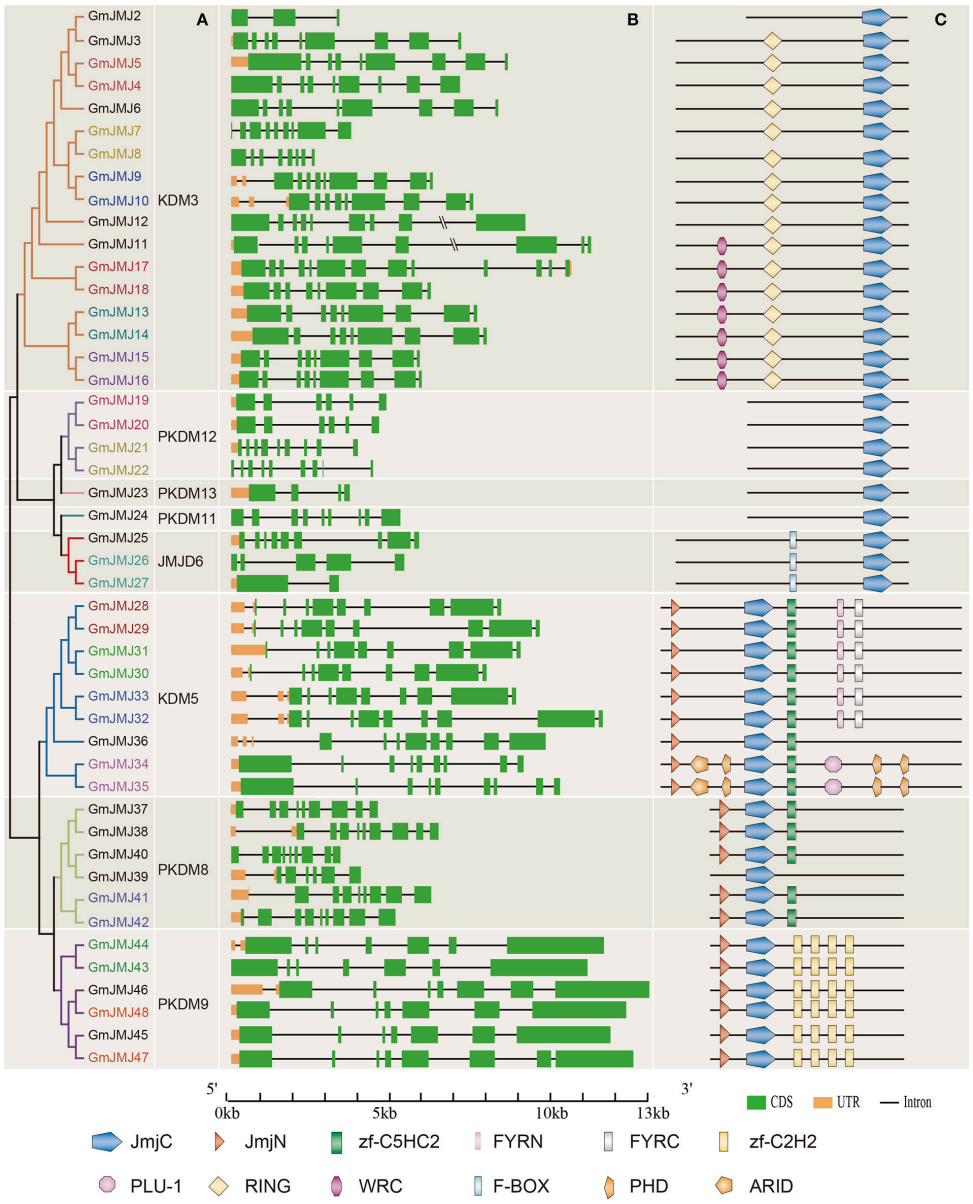
**Phylogenetic analysis, gene structure, and domain architecture of GmJMJs. (A)** Phylogenetic tree construction of GmJMJs based on the JmjC domain amino acid sequences. Name of genes marked in same color are a pair of paralogs. **(B)** Exon/intron structures of *GmJMJs* genes. The black line refers introns, the green box represents exons, and the orange box refers UTR. Over-longed introns were represented with slash–slash. The sizes of exons and introns can be estimated using the scale at the bottom. **(C)** The domain architecture of the full-length JmjC-domain containing proteins. JmjC, Jumonji C domain; JmjN, Jumonji N domain; PHD, plant homeobox domain; ARID, AT-rich interaction domain; zf-C2H2, Zinc finger of C2H2-type; zf-C5HC2, Zinc finger of C5HC2-type; FYRN, “FY-rich” domain N-terminal; FYRC, “FY-rich” domain C-terminal; WRC, Trp, Arg, and Cys domain; RING, (Really interesting new gene) finger domain.

Structural divergence has been very prevalent in duplicate genes and, in many cases, has led to the generation of functionally distinct paralogs (Lynch and Conery, 2000). To better understand the structural diversity of the *GmJMJs* following duplication events, the exon/intron structures (Figure 4B) were compared using Gene Structure Display Server 2.0 (http://gsds.cbi.pku.edu.cn/). Our analysis clearly revealed that most of the paralogs share a similar gene structure. For example, 12 gene pairs (*GmJMJ9/-10, GmJMJ13/-14, GmJMJ15/-16, GmJMJ19/-20, GmJMJ21/-22, GmJMJ28/-29, GmJMJ30/-31, GmJMJ32/-33, GmJMJ34/-35, GmJMJ41/-42, GmJMJ43/-44*, and *GmJMJ47/-48*) were found to have highly consistent gene structures, including the numbers of exons/introns and the length of exons. However, there were some differences in intron lengths and in the 5′ UTR region, which is related to the regulation of expression. For example, *GmJMJ9/-10* and *GmJMJ30/-31* both present a large divergence in the length of their 5′ UTR, implying that a subtle distinction in function in the development and growth of soybean may have appeared between the two paralogs. In addition, four gene pairs (*GmJMJ4/-5, GmJMJ7/-8, GmJMJ17/-18*, and *GmJMJ26/-27*) had greater changes in their structural organization, especially in the numbers of exons.

We also studied the proteins encoded by the *GmJMJs*, using the full-length protein sequences of JmjCs as queries in CDD (Marchler-Bauer et al., 2015), SMART (Letunic et al., 2015), and Pfam (Finn et al., 2016) with default parameters in order to gain more insights into the diversity of the domain architecture, as shown in Figure 4C. These proteins all share a JmjC domain. The JmjN domain was the second most widespread domain, appearing in the majority of members of three groups, KDM5, PKDM8, and PKDM9. This domain, which is not adjacent with JmjC, was identified in the jumonji family (Balciunas and Ronne, 2000), and its interaction with the JmjC catalytic domain was found to be important for Jhd27 (also known as KDM5), a H3K4-specific demethylase in budding yeast (Huang et al., 2010; Quan et al., 2011). In PKDM9, the zf-C2H2 domain, which contains two cysteines and two histidines that coordinate a zinc atom to create a compact nucleic acid-binding domain (Chrispeels et al., 2000), was found in four tandem repeats. Furthermore, another zinc-finger domain, zf-C5HC2, was identified in PKDM8 and KDM5. Three groups, PKDM11, PKDM12 and PKDM13, all have only one domain (JmjC) in their full-length sequence, and can be grouped together as “JmjC domain-only proteins,” in keeping with previous studies (Klose et al., 2006; Lu et al., 2008; Huang et al., 2016). Two-thirds of the members of KDM5 have FYRN and FYRC domains, which may harbor chromatin-binding activity (Lu et al., 2008) or contribute to JmjC function by interacting with other proteins. For example, it has been reported that the functional specificity of AtJMJ14 in flowering time control is based on the specificity of its interaction with transcription factors through the FYRC domain (Ning et al., 2015). Two GmJMJ proteins, GmJMJ34 and GmJMJ35, have the ARID domain (AT-rich interaction domain), which has been implicated in sequence-specific DNA binding (Gregory et al., 1996).

Strangely, we found that not only the gene pairs which share a similar gene structure but also the four pairs which had greater differences in gene structural organization all had consistent domain architectures. For example, *GmJMJ26/-27* share a relatively consistent functional domain in the full-length protein sequences. This implies that although the gene structure of JmjC family may change through evolution, their protein structures and functions were conserved.

### Conserved amino acid residues in active sites of GmJmJ

Fe(II) iron and α-KG are needed as cofactors by JmjC-domain proteins to carry out their demethylase activity (Chen et al., 2006; Huang et al., 2016). A total of five amino acid residues are needed to bind these cofactors; three residues (His188, Glu/Asp190, and His276) bind to the Fe (II) cofactor and two other residues (Thr/Phe185 and Lys206) bind to α-KG. With the aim of clarifying whether the conserved residues interacting with cofactors had diverged among *GmJMJs*, we aligned the domain sequences of JmjC proteins from soybean and *Arabidopsis*.

**Figure 5:**
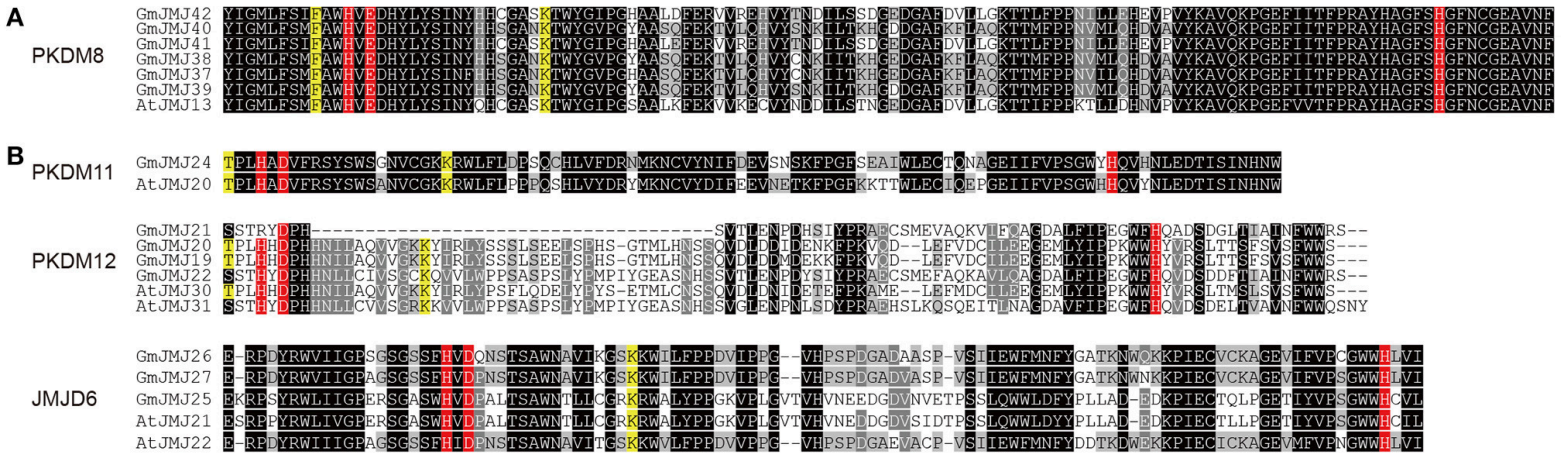
**Two alignment groups (A,B)** of JmjC domain sequences. (Only the representative sequences were shown, other sequences can be seen in Figure S3). The conserved residues compatible with the demethylation activity within the Fe(II) binding site are highlighted in red and those in the αKG binding site are indicated in yellow. The sequences with black, gray, light gray background indicated identical 100%, conservative (75–99%), and block (50–74%) similarity of amino acid residues, respectively.

Based on the alignments, we grouped these proteins into two groups according to amino acids at the conserved sites. The first group, which includes PKDM8, PKDM9, and KDM5, has the conserved amino acids His (H), Glu (E), and His (H) for Fe(II) binding, and Phe (F) and Lys (K) for α-KG binding (Figure 5A), while the second group, which includes JMJD6, PKDM13, PKDM11, PKDM12, and KDM3, has conserved the residues His (H), Asp (D), and His (H) for Fe(II) and Thr (T) and Lys (K) for α-KG (Figure 5B). Both forms are compatible with histone demethylation activity (Lu et al., 2008). In general, most members have conserved residues for interacting with cofactors, although there are some exceptions. For example, a substitution can be seen in the first sites in PKDM12, where Thr (T) was changed into Ser (S) in GmJMJ21/22 and AtJMJ31. However, Thr and Ser have similar physical and chemical properties, so the ability to bind to cofactors may not have changed despite the presence of a different amino acid. Furthermore, the first site to interact with α-KG is absent in JMJD6, which is consistent with findings in rice (Lu et al., 2008). The detection of this change in all three plants, soybean, rice, and *Arabidopsis*, suggests that it may have occurred in the ancestor of these plants and is necessary for their common function. Overall, the high conservation in the interaction sites implies a significant role for these sites in the demethylase activity of the *JmjC* gene family.

### Expression profiles of *JmjC* genes in soybean

**Figure 6:**
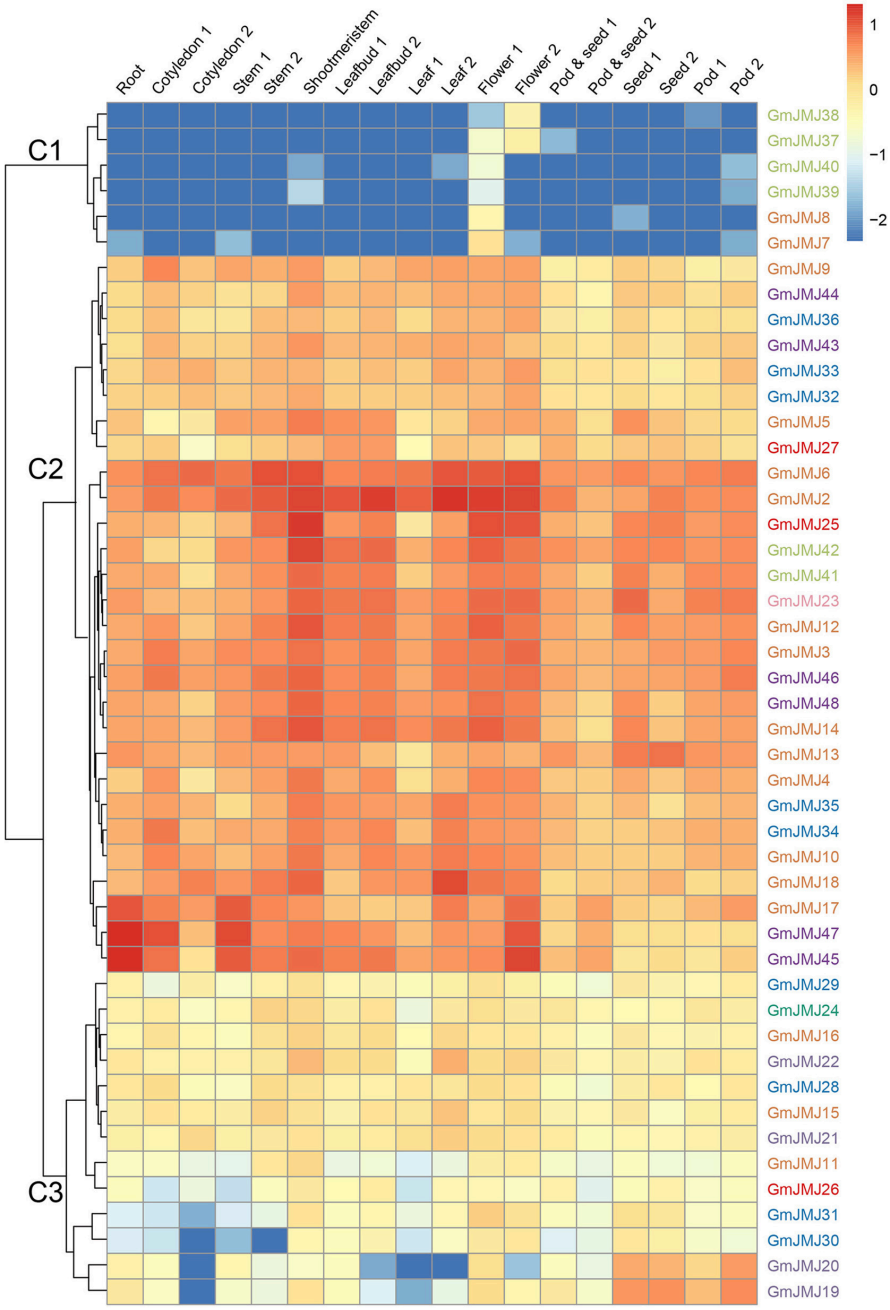
**Heatmaps representing the expression profiles of ***GmJMJs*** in several tissues**. The numbers following the tissues indicated different developmental stages. The color scale on the right indicates expression values, blue indicating low transcript abundance, and red indicating high levels of transcript abundance.

To investigate the tissue-specific expression profiles of *GmJMJs*, transcriptome data (Shen et al., 2014b) were studied in 10 tissues at different developmental stages including roots, cotyledons, stems, shoot meristems, leaf buds, leaves, flowers, pods, pod and seeds, and seeds. As indicated in Figure 6, the expression patterns of the *GmJMJs* can be divided into three clusters, C1–C3. C1 genes show hardly any expression in almost all of the tissues except certain expression in flower, implying that these genes may possess certain specific function in flower after WGD events. C2 can be divided into two subgroups, C2-Sub1 and C2-Sub2, according to their expression levels, with lower expression levels in C2-Sub1 than C2-Sub2. Genes in C3 show a low expression level in all tissues investigated. We found that genes, which belong to the same clade in the phylogenetic tree (Figure 1A), were sometimes dispersed in different clusters based on expression (Figure 6). For example, the four genes in C1 (*GmJMJ37, GmJMJ38, GmJMJ39*, and *GmJMJ40*) grouped together with *GmJMJ41* and *GmJMJ42* in a clade, indicating that they may have been produced through evolution after WGD events, but clustered into two clusters and show different expression patterns, implying they have acquired different functions after the duplication event.

**Figure 7:**
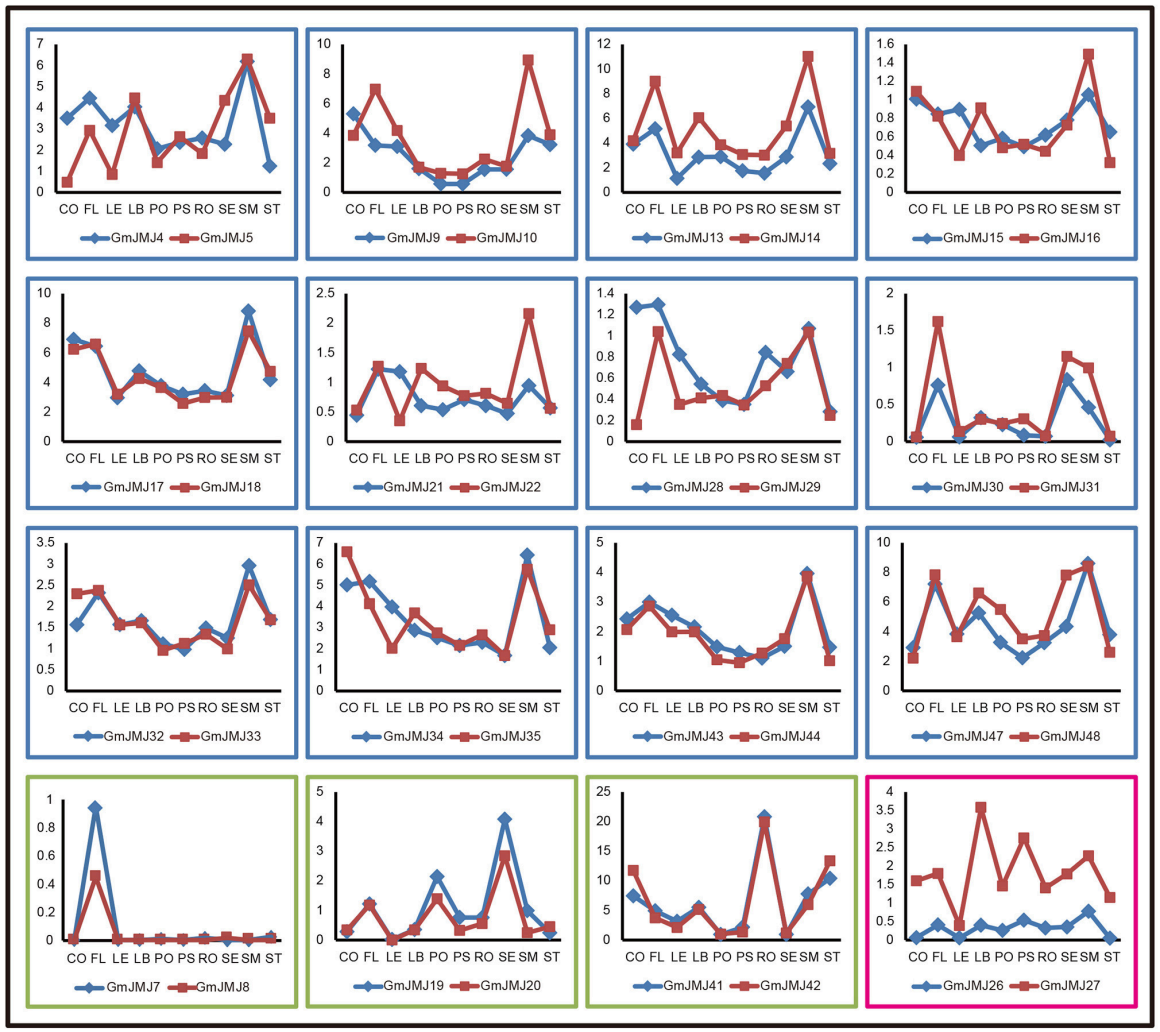
**Three trends of expression patterns of duplicated ***GmJMJ*** gene pairs**. X-axis indicates representative tissues and Y-axis represents scale. The sample used are Cotyledon1(CO), Flower1 (FL), Leaf1(LE), LeafBud1(LB), Pod1(PO), Pod and Seed1(PS), Root1(RO), Seed1(SE), ShootMeristem(SM), Stem1(ST).

In addition, we determined the expression profiles of the recently duplicated *JmjC* gene pairs in 10 tissues. Most of the paralogs generally have the same expression pattern. For further analysis, we divided the duplicated genes into three types based on their detailed expression patterns, shown with a blue, green, and red box in Figure 7. The 12 paralogs in the blue box all have a relatively low expression level in four tissues (pods, podseeds, roots, and seeds) compared with other tissues. These paralogs also show a complex expression pattern in another six tissues examined, but almost all have high expression in the flower, leaf, and shoot meristem. For example, the two copies *GmJMJ4* and *GmJMJ5* both have high expression in flowers, leaf buds and shoot meristems. The expression pattern of these genes can therefore be pictured as similar to the shape of the letter “M.” The three paralogs in the green box only show high expression in one organ, such as the flower, seed, and root. For instance, *GmJMJ7/-8 and GmJMJ41-42* both have a high expression level in flower and roots, respectively. The third box contains only one paralog pair (*GmJMJ26* and *GmJMJ27*), and the expression level of one copy (*GmJMJ27*) is higher than the other copy (*GmJMJ26*) in all tissues. Overall, the paralogs show conserved expression profiles, demonstrating that the *JmjC* gene family has conserved its functions through duplication events.

## Discussion

As histone demethylases, JmjC domain-containing proteins play essential roles in histone modification, which is a significant part of epigenetics (Klose et al., 2006; Chen et al., 2011). To date, many efforts on the *JmjC* gene family have been undertaken to elucidate their evolutionary history in a wide variety of plant species, such as *Arabidopsis* (Lu et al., 2008; Zhao et al., 2015), rice (Lu et al., 2008; Zong et al., 2013), and *Fragaria vesca* (Gu et al., 2016). However, little is known about the *JmjC* gene family in soybean. In this study, we performed a comprehensive analysis of *GmJMJs*, including their phylogenetic relationships, gene structure, domain architecture, chromosome location, duplication patterns, and expression profiles.

### Phylogeny and domain architectures of JmjCs in soybean

In total, 48 *JmjC* genes were identified in the soybean genome, which is larger than other model plants or the other two legumes examined. The number of JmjCs from each species in each group is summarized in Table 1. In most groups, there is still a larger number of JmjCs from soybean than any other species, indicating that these groups may have different evolutionary history among the five species. PKDM11 and PKDM13, two exception groups, both of which including a single gene from each species, may have no duplication or loss after divergence from *Arabidopsis*. The phylogenetic analysis of *JmjC* genes among five plant species showed that each group contains *JmjCs* from all species investigated, four eudicots and one monocot, revealing that the JmjC family may have already existed before the divergence of these two lineages. And combining the phylogeny with the time estimation, 16 *GmJMJs* pairs, formed by the most recent WGD of soybean, were identified.

Although many *GmJMJ* genes were produced by WGD events, there has been little differentiation in their gene structure, domain architecture and the conserved residues acting with cofactors. Another interesting phenomenon is that even outside the conserved coactive-sites we found GmJMJs to have higher amino acid similarity with AtJMJs from the same sub-cluster rather than with GmJMJs from other sub-clusters even though these genes were all grouped in one clade. For example, in PKDM8, GmJMJ41 and GmJMJ42 have the same residues as AtJMJ13 around the Lys (K) site, a Cys (C) and Ser (S), whereas GmJMJ37/-38/-39/-40 have different residues. The unconserved sites around the coactive-sites indicate that the JmjCs may use different ways to bind to the co-factors. However, further evidence is needed to demonstrate and understand this mechanism.

### WGDs contributed to *JmjC* gene expansion in *Glycine max*

WGD/segmental duplication and tandem duplication might lead to duplicated gene pairs on the DNA level. Previous studies have shown that the soybean genome has undergone two rounds of WGD (Schmutz et al., 2010). In this study, we have demonstrated that 16 soybean paralogous pairs derived from the second WGD, which suggests that the WGD duplication might be the main mechanism of *JmjC* gene family expansion and functional diversity during the evolution of soybean. This result is consistent with some other gene families not only *AAT* gene family (Cheng et al., 2016), GST supergene family (Liu et al., 2015), and receptor-like kinase genes (Zhou et al., 2016) in soybean, but also SET domain family in *Populus trichocar* (Lei et al., 2012; Zhang and Ma, 2012), and 14-3-3 family genes and AP2/ERF superfamily in *M. truncatula* (Qin et al., 2016; Shu et al., 2016). And in *Arabidopsis*, previous studies have proposed that more than 90% regulatory genes increased due to WGD (Maere et al., 2005). However, the dispersed duplications and retro-transpositions played the most important role in the evolution of *JmjC* genes in *F. vesca* (Gu et al., 2016). Furthermore, KDM3 group is preferentially expanded in the soybean genome compared to other groups, consistent with *F. vesca* (Gu et al., 2016), indicating that KDM3 group genes may have evolved to meet some unique regulatory needs.

### Expression profiles of *JmjC* genes and functional diversity of duplicated pairs in soybean

In angiosperms and vertebrates, both of the *JmjC* and *SET* genes, maintaining homeostasis of the histones methylation, are the key regulators of chromatin structure, suggesting that the epigenetic modulation playing an important role in regulation of gene expression in developmental stages and responses to abiotic stresses (Lei et al., 2012; Zhang and Ma, 2012; Qian et al., 2015). We investigated the expression profiles of *JmjC* genes using public expression data and found that most *JmjC* genes are widely expressed (Figure 6), indicating that these genes, remained after WGD/segmental events, are likely functional. To further elucidate whether functional differentiation has occurred after the WGD event, we analyzed the expression patterns between the duplicated pairs (Figure 7). All the expression patterns of 16 duplicated gene pairs can be classed into three types according to their tendency in each tissues detected. The first type is that two copies have the complicated expression patterns in different tissues. We could hypothesize possible functions of *GmJMJs* by coupled their expression patterns with the functions of their *Arabidopsis* orthologs. For example, *AtJMJ24*, the ortholog of *GmJMJ17* and *GmJMJ18* in *Arabidopsis*, is a histone H3K9 demethylase (Lu et al., 2008). *AtJMJ24* has been proved to promote basal level transcription of endogenous silenced loci by counteracting H3K9me (Deng et al., 2015). Therefore, *GmJMJ17* and *GmJMJ18* might have the functions similar to *AtJMJ24* in reinforcing the silence. The second type showed that both copies have high expression in one organ, such as the flower, seed, and root. The third type is that one duplicate was expressed at higher levels than the other one nearly in all tissues, implying that the former one has stronger function than the latter, and implying that it may play important roles in regulating broad developmental or reproductive stages. Above all, the expression patterns among the duplicated pairs are relatively conserved, suggesting little functional differentiation has occurred following the WGD event. However, some *JmjC* genes were specific to soybean, for example, *GmJMJ4* and *GmJMJ5*. All of these genes have abundant transcripts in soybean and are expressed at different levels in different tissues. These results indicate that in *Arabidopsis* their counterparts may be lost and the functions might have been performed by other genes.

## Conclusions

Here, we performed comprehensive and evolutionary analyses of *JmjC* gene family in soybean, and provided detailed information on its members. A total of 48 putative *JmjC* genes were identified in the soybean genome, which represented non-random across all soybean genome chromosomes and majority of them expanded from WGD/segmental duplication rather than the dispersed duplications. The exon/intron compositions and domain arrangements were considerably conserved among members in the same groups or subgroups. Many duplicated genes present similar expression patterns in soybean tissues detected implying functional conservation. The close phylogenetic relationship between *GmJMJs* and *AtJMJs* in the same subgroup provided insights into their putative functions. Taken together, all of these results provided valuable clues in future efforts to identify specific gene functions of this gene family and gene diversity among different genotype of soybean and other plants in *Leguminosae*.

## Author contributions

LW and LZ designed the research; YH, XL, and LW performed phylogenetic analysis and wrote the manuscript; LC, YL, and HW annotated the *JmjC* genes on chromosomes and calculated the duplication date; LC, DK, and HY analyzed the expression data.

### Conflict of interest statement

The authors declare that the research was conducted in the absence of any commercial or financial relationships that could be construed as a potential conflict of interest.

## Supplementary material

Figure S1 **Phylogenetic relationship of JmjC-domain containing proteins from five plant species by using the JmjC domain alone**.

Figure S2 **Phylogenetic relationships of ***GmJMJs*****.

Figure S3 **Alignment of JmjC domain sequences**. The conserved residues compatible with the demethylation activity within the Fe(II) binding site are highlighted in red and those in the αKG binding site are indicated in yellow. The sequences with black, gray, light gray background indicated identical 100%, conservative (75–99%), and block (50–74%) similarity of amino acid residues, respectively.

Table S1 **Protein sequences of Arabidopsis, ***Oryza sativa*** JmjC domain-containing protein used as blast queries**.

Table S2 **The domain sequences used in the phylogenetic trees**.

Table S3 **The expression data of ***GmJmjC*** genes in various organs**.

Table S4 **Detailed information of soybean ***JmjC*** family genes**.

Table S5 **The duplication types of ***GmJmjC*** genes calculating by MCScanX program**.
