# Supplementary figures and images for: Genome-Wide Analysis of Soybean JmjC Domain-Containing Proteins Suggests Evolutionary Conservation Following Whole-Genome Duplication
Source: Front Plant Sci. 2016 Dec 5;7:1800. doi: 10.3389/fpls.2016.01800 (PMC5136575; doi:10.3389/fpls.2016.01800)

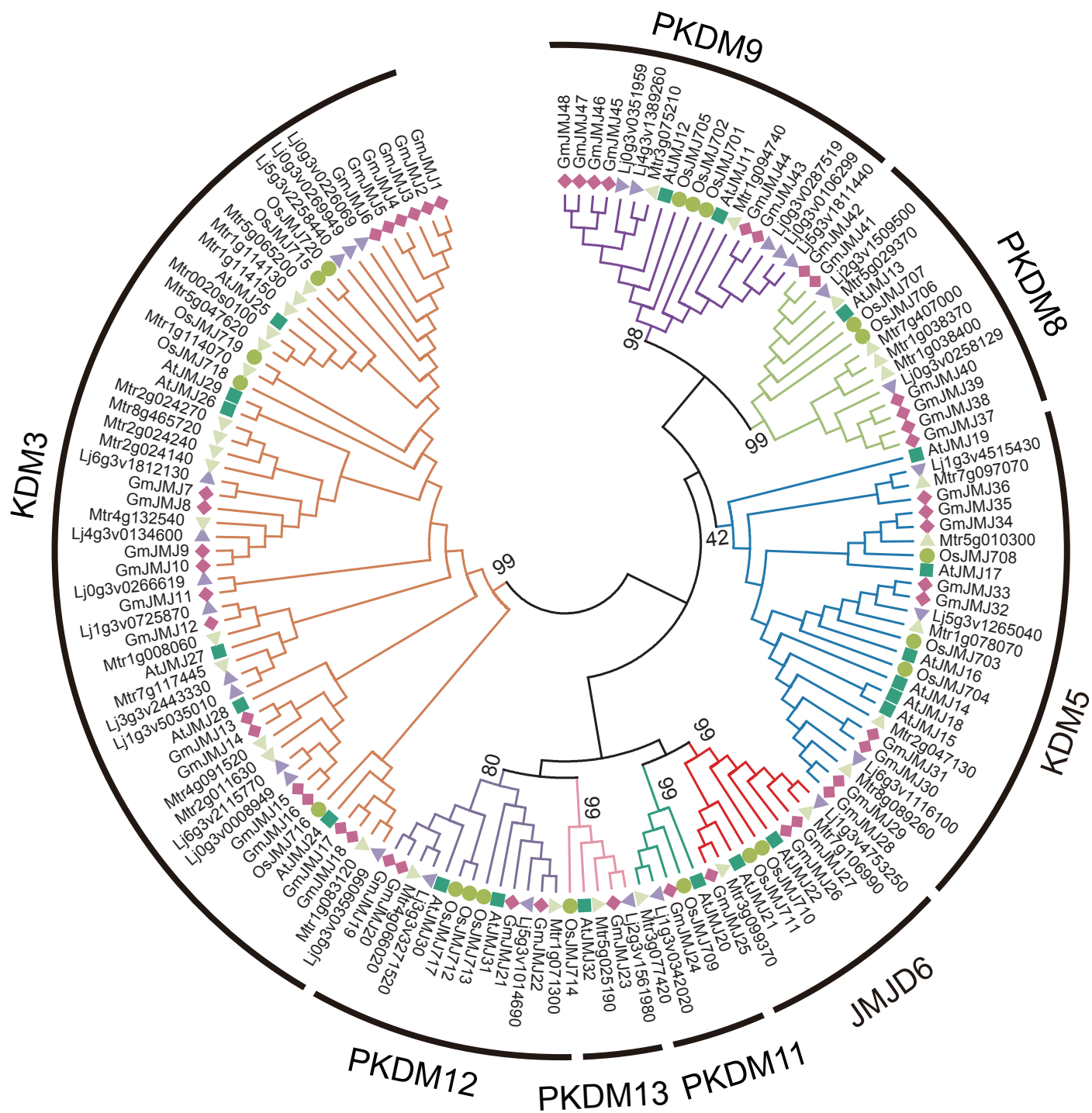

Supplement: Figure S1 — Phylogenetic relationship of JmjC-domain containing proteins from five plant species by using the JmjC domain alone. [file Image1.PDF]

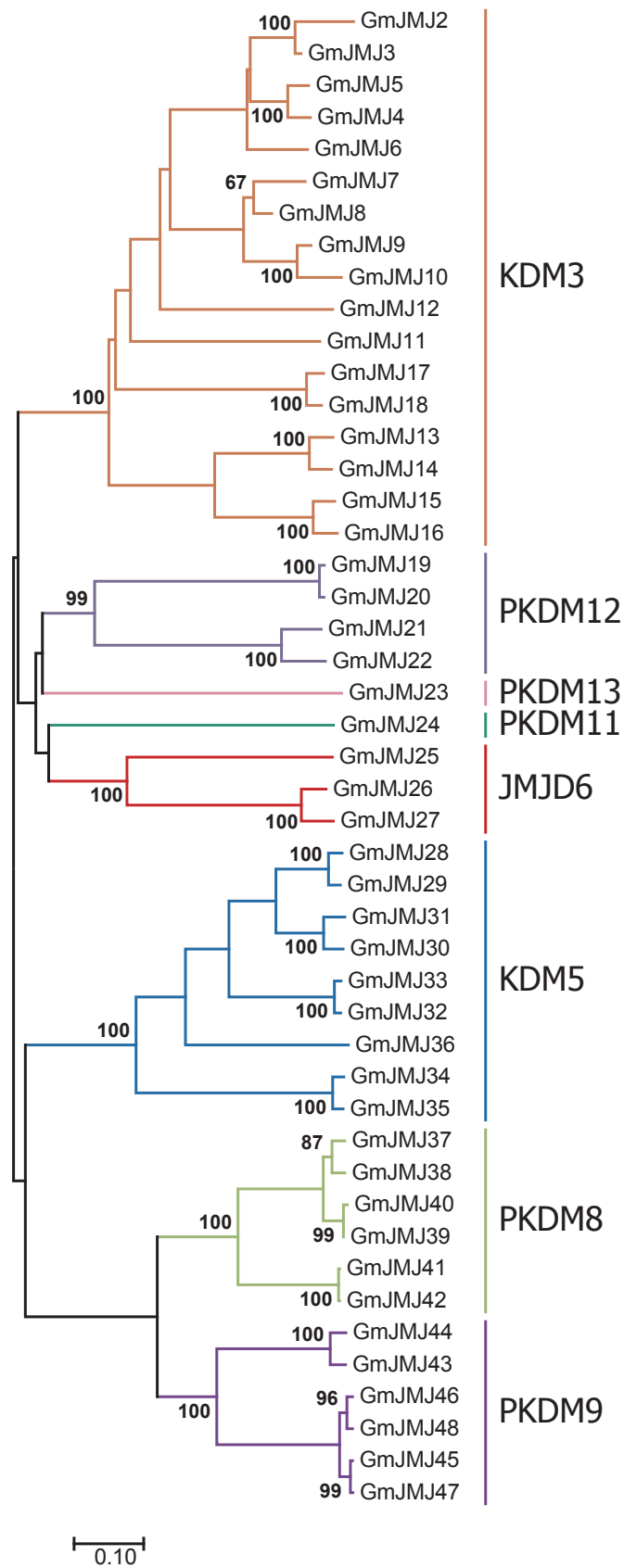

Supplement: Figure S2 — Phylogenetic relationships of GmJMJs. [file Image2.PDF]
