# Supplementary material for: Genome-Wide Analysis of Soybean JmjC Domain-Containing Proteins Suggests Evolutionary Conservation Following Whole-Genome Duplication
Source: Front Plant Sci. 2016 Dec 5;7:1800. doi: 10.3389/fpls.2016.01800 (PMC5136575; doi:10.3389/fpls.2016.01800)
Supplement: Figure S3 — Alignment of JmjC domain sequences. The conserved residues compatible with the demethylation activity within the Fe(II) binding site are highlighted in red and those in the αKG binding site are indicated in yellow. The sequences with black, gray, light gray background indicated identical 100%, conservative (75–99%), and block (50–74%) similarity of amino acid residues, respectively. [file Image3.PDF]

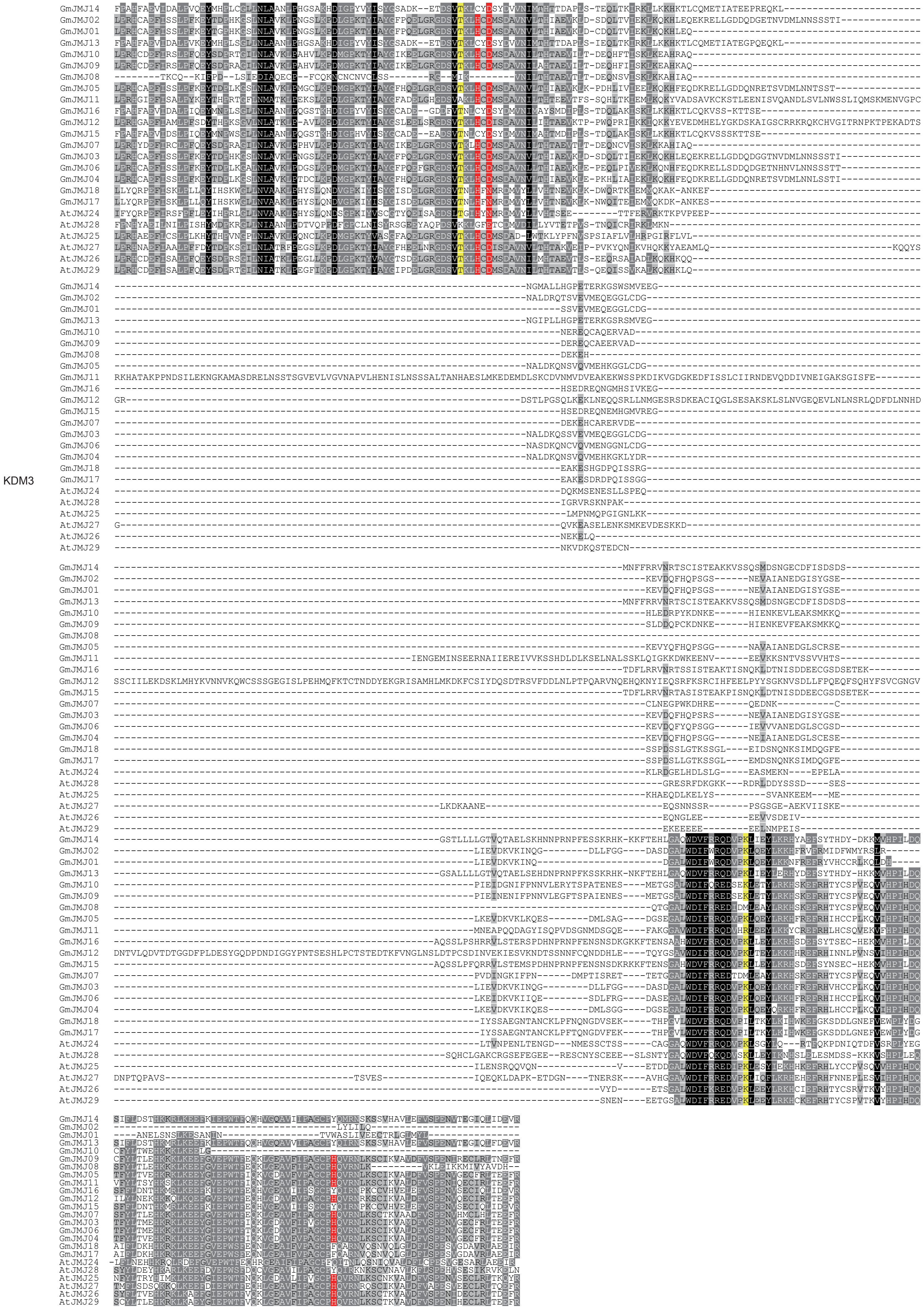
KDM3

PKDM13

```
GmJMJ23 TSFHKDHYENLYAVVTGEKHFLLLPPTDVHRLYIRDYPAATYSYSSDTGEFDLELEKPTRYVPWCSVDPYPSLETMDNEMTKFPLYFNGPRPFECTVKAGEVLYLPSMWFHHVRQGVDDGGLTIAVNYWYDMQFD
AtJMJ32 TSFHKDHYENLYAVVSGEKHFLLLPPTDVHRLYIEQYPAANYSYHRDTDAFKLEVEEPVRHVPWSSVDPYPSPEKEASERLKFPLFFDGPKPFHCTVKAGEVLYLPSMWFHHVSQTPGDGGYTIAVNYWYDMQFD
```

PKDM9

```
GmJMJ48 YVAMLFSWFAWHVEDHDLHSLNYLHMGAGKTWYGVPRDAAVAFEEVVRVHGYGGEINPL-VTFATLGEKTTVMSPEVFISAGVPCCRLVQNAGEFVVTFPRAYHSGFSHGFNCGEAANI
GmJMJ46 YVAMLFSWFAWHVEDHDLHSLNYLHMGAGKTWYGIPRDAAVAFEEVVRVHGYGGEINPL-VTFAILGEKTTVMSPEVFISAGVPCCRLVQNAGEFVVTFPRAYHTGFSHGFNCGEAANI
GmJMJ47 YVAMLFSWFAWHVEDHDLHSLNYLHMGAGKTWYGVPRDAAVAFEEVVRVHGYGGEINPL-VTFATLGEKTTVMSPEVLISAGVPCCRLVQNAGEFVVTFPRAYHTGFSHGFNCGEAANI
GmJMJ45 YVAMLFSWFAWHVEDHDLHSLNYLHMGAGKTWYGVPRDAAVAFEEVVRVHGYGGEINPL-VTFAILGEKTTVMSPEVFISAGVPCCRLVQNAGEFVVTFPRAYHTGFSHGFNCGEAANI
GmJMJ44 YIGMLFSWFAWHVEDHELHSMNFLHTGSSKTWYAVPGDYAFAFEEVIRTEGYSGNIDHL-AALKLLGEKTTLLSPEVIVASGIPCCRLTQHPGEFVVTFPRAYHVGFSHGFNCGEAANF
GmJMJ43 YIGMLFSWFAWHVEDHELHSMNFLHTGSSKTWYAVPGDYAFAFEEVIRTEGYSGNIDHLGICLKLLGEKTTLLSPEVIVASGIPCFRLTQHPGEFVVTFPRAYHVGFSHGFNCGEAANF
AtJMJ12 YVAMMFSWFAWHVEDHDLHSLNYLHMGAGKTWYGVPKDAALAFEEVVRVHGYGEELNPL-VTFSTLGEKTTVMSPEVFVKAGIPCCRLVQNPGEFVVTFPGAYHSGFSHGFNFGEASNI
AtJMJ11 YIGMLFSWFAWHVEDHELHSMNYLHTGSPKTWYAVPCDYALDFEEVIRKNSYGRNIDQL-AALTQLGEKTTLVSPEMIVASGIPCCRLVQNPGEFVVTFPRSYHVGFSHGFNCGEAANF
```

KDM5

```
GmJMJ36 KIHMGMCFSPLNWKVEEHHLYSLYYVHLGEPKVWYGIPGKFAINFETIWKKYLPDLQAGQPDMHDNMVMQLSCSILKAEGIPVYRCIQYPREFVLVFPGSYHSGFDCGFNCSEAVSF
GmJMJ31 WLYVGMCFSSFCWHVEDHHLYSLNYLHWGDPKVWYGVAGSHAPGLEDAMRKHLPDLFEEQPNLLNELVTQLSPSILKSEGVPVHRTIQHSGEFVVTFPRAYHCGFNCGFNCAEAVNV
GmJMJ30 WLYVGMCFSSFCWHVEDHHLYSLNYLHWGDPKVWYGIPGSHAPGLEDAMRKHLPDLFEEQPNLLNELVTQLSPSVLKSEGVPVHRTVQHSGEFVVTFPRAYHCGFNCGFNCAEAVNV
GmJMJ29 WLYVGMCFSSFCWHVEDHHLYSLNYLHWGDPKVWYGVPGSHAAALEKVMRKHLPDLFEEQPNLLNDLVTQFSPSILKSEGVPVYRTVQHSGEFVITFPRAYHAGFNCGFNCAEAVNV
GmJMJ33 WLYVGMCFSSFCWHVEDHHLYSLNYMHWGAPKMWYGVPGKDACKLEEAMRKHLPELFEEQPDLLHKLVTQLSPSILKSKGVPVYRCIQNPGDFVLTFPRAYHSGFNCGFNCAEAVNV
GmJMJ28 WLYVGMCFSSFCWHVEDHHLYSLNYLHWGDPKVWYGVPGSHAAALEKVMRKHLPDLFEEQPNLLNDLVTQFSPSILKSEGVPVYRTVQHSGEFVITFPRAYHAGFNCGFNCAEAVNV
GmJMJ32 WLYVGMCFSSFCWHVEDHHLYSLNYLHWGAPKMWYGVPGKDACKLEEAMRKHLPELFEEQPDLLHKLVTQLSPSILKSKGVPVYRCIQNPGDFVLTFPRAYHSGFNCGFNCAEAVNV
GmJMJ35 WLYIGMLFSSFCWHFEDHCFYSMNYLHWGEAKCWYSVPGSQASAFEKVMKNSLPDLFDAQPDLLFQLVTMLNPSVLQENGVPVYSILQEPGNFVITFPRSYHGGFNLGLNCAEAVNF
GmJMJ34 WLYIGMLFSSFCWHFEDHCFYSMNYLHWGEAKCWYSVPGSQATAFEKVMKSSLPDLFDAQPDLLFQLVTMLNPSVLQENGVPVYSILQEPGNFVITFPRSYHGGFNLGLNCAEAVNF
AtJMJ16 WLYIGMCFSSFCWHVEDHHLYSLNYMHWGAPKLWYGVGGKDAVKLEEAMRKHLPDLFEEQPDLLHKLVTQLSPSKLKTAGVPVHRCVQHAGEFVLTFPRAYHAGFNSGFNCAEAVNV
AtJMJ18 WLYVGMCFSSFCWHVEDHHLYSLNYHHFGEPKVWYGVPGSNATALEKAMRKHLPDLFEEQPDLLHGLVTQFSPSILKDEGVQAYRVVQNSGEYVLTFPRAYHAGFNCGFNCAEAVNV
AtJMJ19 RLSVGMCLSSQFWKSEKERLYSLCYLHVGAPRVWYSVAGCHRSKFKAAMKSFILEMSGEQPKKSHNPVMMMSPYQLSVEGIPVTRCVQHPGQYVIIFPGSYYSAFDCGFNCLEKANF
AtJMJ15 WLYVGMCFSTFCWHVEDNHLYSLNYHHFGEPKVWYGVPGSHATGLEKAMRKHLPDLFDEQPDLLHELVTQFSPTILKNEGVPVYRAVQNAGEYVLTFPRAYHSGFNCGFNCAEAVNV
AtJMJ14 WLYVGMCFSTFCWHVEDHHLYSMNYLHTGDPKVWYGIPGNHAESFENVMKKRLPDLFEEQPDLLHQLVTQLSPRILKEEGVPVYRAVQRSGEFILTFPKAYHSGFNCGFNCAEAVNV
AtJMJ17 WLYLGMLFSSFCWHFEDHCFYSVNYLHWGEAKCWYGIPGSAASAFEKVMRKTLPDLFDAQPDLLFQLVTMLSPTVLQENKVPVYTVLQEPGNFVITFPKSFHAGFNFGLNCAEAVNF
```
